# Mechanics of lipid bilayer junctions affecting the size of a connecting lipid nanotube

**DOI:** 10.1186/1556-276X-6-421

**Published:** 2011-06-14

**Authors:** Roger Karlsson, Michael Kurczy, Richards Grzhibovskis, Kelly L Adams, Andrew G Ewing, Ann-Sofie Cans, Marina V Voinova

**Affiliations:** 1 Department of Chemistry, University of Gothenburg, Kemivägen 10, 41296 Gothenburg, Sweden; 2 Department of Chemical and Biological Engineering, Chalmers University of Technology, 41296 Gothenburg, Sweden; 3 Applied Mathematics, University of Saarland, 66121 Saarbrücken, Germany; 4 Department of Chemistry, Penn State University, 104 Chemistry Building, University Park, PA 16802, USA; 5 BioNano Systems Laboratory, Institute for Microtechnology and Nanoscience, Chalmers University of Technology, 41296 Gothenburg, Sweden

## Abstract

In this study we report a physical analysis of the membrane mechanics affecting the size of the highly curved region of a lipid nanotube (LNT) that is either connected between a lipid bilayer vesicle and the tip of a glass microinjection pipette (tube-only) or between a lipid bilayer vesicle and a vesicle that is attached to the tip of a glass microinjection pipette (two-vesicle). For the tube-only configuration (TOC), a micropipette is used to pull a LNT into the interior of a surface-immobilized vesicle, where the length of the tube *L *is determined by the distance of the micropipette to the vesicle wall. For the two-vesicle configuration (TVC), a small vesicle is inflated at the tip of the micropipette tip and the length of the tube *L *is in this case determined by the distance between the two interconnected vesicles. An electrochemical method monitoring diffusion of electroactive molecules through the nanotube has been used to determine the radius of the nanotube *R *as a function of nanotube length *L *for the two configurations. The data show that the LNT connected in the TVC constricts to a smaller radius in comparison to the tube-only mode and that tube radius shrinks at shorter tube lengths. To explain these electrochemical data, we developed a theoretical model taking into account the free energy of the membrane regions of the vesicles, the LNT and the high curvature junctions. In particular, this model allows us to estimate the surface tension coefficients from *R*(*L*) measurements.

## Background

Membrane tethers have been studied extensively over the past 40 years [1-11]. These structures, also called membrane nanotubes, were observed during fluid shear deformation of live cells attached to a substrate. As these cells were dislodged, membranous tethers remained attached to the surface displaying both the fluid and the elastic properties of the membrane [1,2]. Following this work many naturally forming membrane nanotubes have been identified [7-10]. For example, membrane nanotubes have been shown to exist within the cell, notably in the trans golgi network [10]. Here, lipid and protein cargo destined for various destinations throughout the cell are sorted and pinched off from the tubular membrane of the network. It has also been reported that cells have the ability to use membrane nanotubes for the exchange of organelles [7], and this exchange has interestingly even been recognized between different cell types [8]. Thus, these tethers, which were first observed following a dramatic manipulation, have been shown to be a common occurrence in biology.

**Figure 1:**
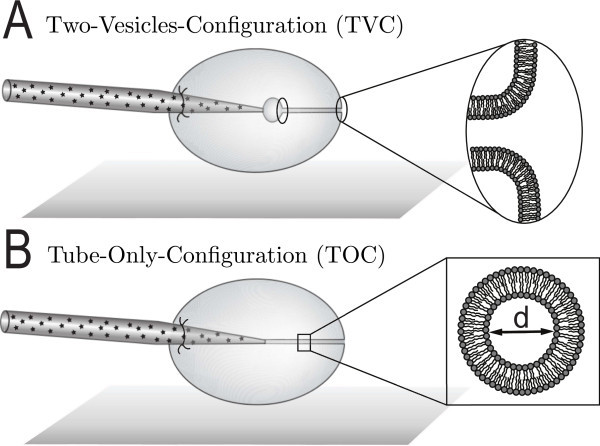
**Experimental configurations**. Sketches of the geometries of the large unilamellar vesicles interconnected with a common LNT; **(A) **the "two-vesicle" configuration, where the LNT is connected between the mother vesicle and a small daughter vesicle inside of the mother vesicle, **(B) **the "tube-only" configuration where the LNT is connected between the tip of a glass pipette and the giant unilamellar vesicle.

Following their initial discovery, the lipid membrane nanotubes (LNTs) have been created artificially in several model membrane systems. By attaching a bead or a micropipette to a point on the membrane and applying a localized mechanical force to the bilayer surface it has been shown that a lipid tether can be pulled from the vesicle membrane [3-5,11]. The size of the structure is a result of the interplay between the curvature elasticity effects maintaining the original geometry and the membrane tension [12]. Tether pulling experiments can be used for estimations of tube diameters. By measuring the forces required for pulling a tube, the diameter of the LNTs were estimated to be 50-200 nm [13]. From a tube coalescence method [14] and video pixel analysis of accumulated fluorescence images as well as from micrographs obtained with differential interference contrast optics [5], the LNT diameters were determined to be in the range of 100-300 nm [13]. To complement these methods, we developed an electrochemical method to monitor the diffusion of electroactive molecules through the LNT, thus allowing the LNT diameter to be measured as a function of nanotube length [11]. The method relies on the formation of a vesicle-LNT network by using a micropipette technique [5,15]. The micropipette-assisted vesicle-LNT network formation allows us to create complex systems of vesicles interconnected by LNTs, including a so-called inward configuration where a small daughter vesicle is created inside a larger mother vesicle, the two vesicles being connected by a LNT [6] (see Figure 1A). During network formation, the LNT is pulled with a micropipette to the interior of the vesicle and thus the opening of the tube faces outward to the exterior of the vesicle. This makes it possible to monitor the diffusion of a marker molecule from the micropipette, through the tube, and out of the nanotube opening. The concentration of the molecules measured at the opening of the LNT is directly related to the inner diameter of a LNT of determined length [11]. In this article we use the electrochemical method for monitoring the size of a nanotube attached directly to the micropipette in the configuration we refer to as the tube-only configuration (TOC) (see Figure 1B).

Additionally, by inflating a small ("daughter") vesicle at the tip of the micropipette, the diameter of a nanotube placed in between the inner vesicle and membrane of the outer vesicle can be examined in a configuration here called two-vesicle configuration (TVC) (see Figure 1A). The measurements show that there is a reduction in tube diameter at shorter length, and the effect appears to be more pronounced in the TVC. In this work we suggest a geometrical model based on direct minimization of the Helfrich's functional for the system of lipid vesicles linked to a LNT via junctions of specific geometry. This new model presents a unified quantitative analysis of TOC and TVC and explains why the length of the LNT in the TVC is twice as high as in TOC for a given radius. Furthermore, the model has just two parameters, which can be chosen to fit the experimental data on monitoring of the size of the LNT. This allows for identifying the contribution of the surface tension to the free elastic energy of the system. This low-tension term has been neglected in the related publication [11], where a phenomenological description of the system was suggested and only a qualitative consistency with experimental data was obtained.

## Experimentals

### Materials and methods

Surface-immobilized giant unilamellar soybean liposomes (SBL) were made from soybean polar lipid extract (Avanti Polar Lipids, Alabaster, AL), as previously described [5,6,11,15]. An injection pipette pulled with a commercial pipette puller (Model PE-21, Narishige Inc., London, UK) and was back-filled with a 50 mM catechol solution. The pipette was then electro-inserted into the unilamellar liposome with the aid of a voltage pulse generated relative to a 5 *μ*m counter electrode (ProCFE from Dagan Corp, Minneapolis, MN), which was placed on the opposite side of the liposome from the injection pipette. Carbon fiber working electrodes were fabricated in house and have been described elsewhere [11]. Working electrodes were held at +800 mV versus a silver/silver chloride reference electrode (Scanbur, Sweden). All measurements were made using an Axon 200B potentiostat (Molecular Devices, Sunnyvale, CA).

### Nanotube radius measurements and calculations

The flux of catechol through the nanotube was measured using carbon fiber amperometry. A 5 *μ*m carbon fiber microelectrode was placed at the nanotube-liposome junction. The nanotube was then either lengthened or shortened by manipulating the injection pipette. After the new length was obtained, the current was allowed to stabilize and was then recorded. This process was repeated several times for each liposome resulting in a series of electrochemical measurements for tubes of different lengths. The electrode was then removed from the nanotube-liposome junction and allowed to reach a steady current to establish a baseline. The difference in measured current for a nanotube versus this background together with the length of the nanotube was then used to compute the diameter of the nanotube based on the previously derived relationship(1)

where *R *is the radius of the nanotube of a given length *L*, Δ*i *is the change in measured current with respect to the background, *n *is the number of moles of electrons transferred per mole of redox species (for catechol, this is equal to 2), *F *≈ 96 485.34 C/mol is Faraday's constant, *D *= 7.0 *× *10*^-^*^6 ^cm^2^/s is the diffusion coefficient of the selected redox species (catechol). Δ*C *is the change in concentration of catechol over the nanotube length and is equal to the concentration of electroactive species in the pipette assuming that the concentration at the electrode surface is zero (in our experiments Δ*C *= 50 mM).

The results for the tube radius deduced from the simultaneous measurement of electrochemical current and the tube length by using formula (1) are presented in this study. In comparison with our previous publication [11], a wider range of the length *L *of the tube is presented for the TOC configuration.

## Theoretical approach

### The system under consideration

In the first system (Figure 1A), a mother vesicle contains a small daughter vesicle on the inside with a common LNT connecting the two compartments. In the second case (Figure 1B), the lipid tube is pulled to the inside of the vesicle and is directly fixed to the tip of the micropipette. Also, there is a source of lipid attached to the mother vesicle wall. The presence of lipid source means that the surface tension is low. We model the membrane as a two-dimensional surface Γ. Its free elastic energy written in the form of Helfrich functional [16] reads(2)

Here *H *is the mean curvature of the surface, *C*_0 _is the spontaneous curvature which is determined by the specific chemical composition of the membrane, *k *is the coefficient of membrane bending, *σ *is the coefficient of membrane surface tension. The equilibrium shape of the membrane with pulled cylindrical tubule can be found from minimum of the functional

where *f *is the force needed to pull the lipid tube of length *L *[12]. In the case, when the junctions are not taken into account, the interplay between membrane bending *k *and membrane tension *σ *produces variability in tubule radius and the force *f*_0_(3)

where *f*_0 _is the force needed to hold the tube of radius *R*_0 _at a fixed position [12]. However, it was shown that for lipid vesicles interconnected with LNTs, either pulled outward from the vesicle wall [5,15] or inward into the vesicle interior [11,17], the neck elements (the junctions between the lipid tube and the vesicle body) also contribute to the total free energy of the membrane. Below we consider a theoretical model based on the Helfrich functional to find the equilibrium shape of the membrane accounting for the junctions of the specific geometry. By comparing the results of numerical computations with experimental data, we are able to determine the tension in the LNT after fitting the experimental data with the geometrical model described below.

### The geometrical model

When the inner vesicle or the junction between the micropipette and the nanotube is subjected to the translation movement along the LNT axis, the length of the tube is changed (increasing or decreasing its value in a controlled way, which can be monitored under the microscope). During these manipulations the radius of the tube adapts to minimize the Helfrich's free energy (2) with *C*_0 _= 0, as we neglect any contribution from spontaneous curvature.

**Figure 2:**
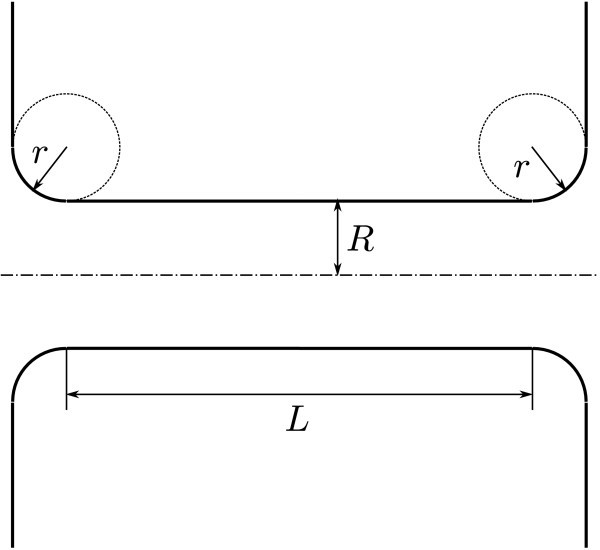
**Schematics of the geometry of the tube-junctions**.

We assume that the shape of LNT can be approximated by a cylindrical surface of radius *R *and length *L*. Since radii of both vesicles are much larger than the tube radius, the junctions between the cylinder and vesicles are modelled by toroidal surfaces with the inner radius *R *+ *r *and crossection radius *r *(Figure 2). In the TOC, when the inner vesicle is not present, only one junction is considered. Although the junction between the micropipette and the tube contributes to the total free energy, it is assumed that this contribution does not depend on the tube radius *R *and, thus, the corresponding term vanishes after the variation. In these settings, the radius-dependent part of the free energy is given by the expression:(4)

where  and(5)

The toroidal part of the surface can be parametrised by (5) due to translation invariance of the energy functional (4). The multiplier *ν *assumes the value 1 for TOC and 2 for TVC to represent both junctions.

In Equation 4, *L*, *r*, *σ *are fixed parameters while the radius of the tube *R *is adjusted to satisfy(6)

The variation (6) yields the following relation between the tube length *L *and radius *R*(7)

where .The model parameter *r *as well as  are chosen to obtain the best fit to the experimental data. Assuming that the radius of the tube *R *is much larger than the parameter *r*, the first two terms of the power series expansion for (7) with respect to *r*/*R *can also be used to quantitatively model the measured relation *L*(*R*). This, simplified, form of (7) reads(8)

and allows for expressing *R *as a function of *L*(9)

An important feature of the proposed model is the asymptote  (compared to (3)), which is present in all three relations (7), (8), and (9). As we increase *L*, the radius *R *grows and the energy of the cylindrical part of the surface becomes dominant over the energy of the toroidal junctions. Thus, in the limit case *L → *∞, we obtain the junction free equilibrium value of *R *given by (3).

### Fitting the parameters

For given *K *measurements (*L_i_*, *R_i_*), *i *= 1 ... *K*, we vary  and *r *to minimize(10)

by means of conjugate gradient minimization procedure. Here, the relation *L*(*R*) is given by (7). When the ratio *r*/*R *is small, the approximation (8) can be used instead. In this case, one can also fit (9) to the data by minimizing the functional(11)

The latter method is preferable when the relative measurement error for *R *is greater than the one for *L*.

## Results and discussion

**Figure 3:**
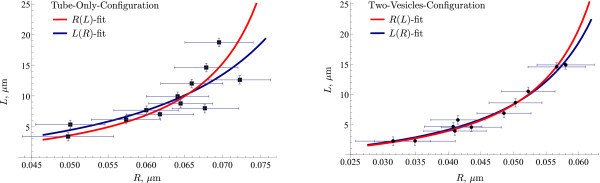
**Comparison of experimental and model results**. The measurement points (shown as markers) and the predictions of the model (solid lines). Parameters for the model predictions were chosen to minimize functionals (11) (red lines) and (10) (blue lines).

When fitting the curve (7) to the dataset for the TVC, the parameter values are *r *≈ 1.7 nm and *σ*/*k *≈ 89 *μ*m*^-^*^2^. The corresponding values for the dataset in the case of TOC are *r *≈ 1.2 nm and *σ*/*k *≈ 54 *μ*m*^-^*^2^. The relation *L*(*R*) with fitted parameters are plotted on Figure 3 (blue curves) together with measured experimental data. As expected, the parameter *r *is much smaller than the radius *R*: *r*/*R <*0.06. Therefore, the simplified form (8) and its inverse (9) can be used for the given range of values of *R*. Fitting the relation (9) to the measurements by minimizing (11) yields *σ*/*k *≈ 98 *μ*m*^-^*^2^, *r *≈ 1.9 nm and *σ*/*k *≈ 72 *μ*m*^-^*^2^, *r *≈ 1.7 nm for TVC and TOC, respectively. The corresponding curves are plotted in Figure 3 in red. The model exhibits good agreement with the empirical data. A rather large scattering of measurement points at high *R *values in the TOC case is reflected as about 20% difference in parameter values when using different approaches to find the best fit. In this case, the values obtained through fitting (9), namely *σ*/*k *≈ 72 *μ*m*^-^*^2^, *r *≈ 1.7 nm have higher reliability.

Our model establishes a connection between the data from TOC and TVC experiments. It follows directly from formula (7), that to reach a given radius *R *of the tube, the length *L*_TVC _of the tube in the TVC experiment must be double of that in TOC arrangement

**Figure 4:**
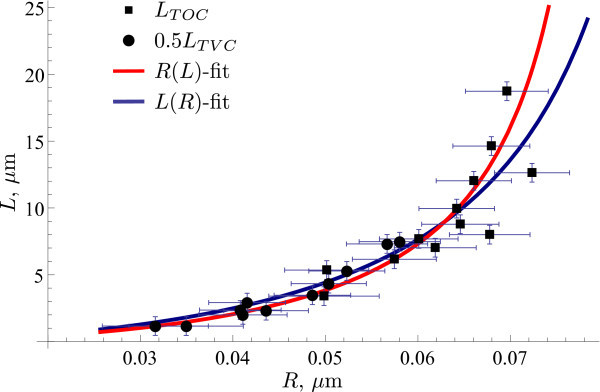
**Comparison of experimental and model results (unified description)**. The measurement points (shown as markers) for both TVC and TOC plotted after dividing the TVC length by two. Parameters for the model predictions were chosen to minimize functionals (11) (red line) and (10) (blue line).

To explore this theoretical prediction, we divide the lengths obtained in the experiment with TVC by two and plot the resulting data set together with the measurements for TOC on Figure 4. The optimal parameters of the model for this, unified, data are *σ*/*k *≈ 55 *μ*m^-2^, *r *≈ 1.2 nm and *σ*/*k *≈ 71 *μ*m *^-^*^2^, *r *≈ 1.6 nm for functionals (10) and (11), respectively. These values are similar to ones for the TOC case since this portion of the data is more disperse and has much greater contributions to functionals (11) and (10) when compared to the data for the TVC case. Figure 4 also shows that the measurements are in agreement in the region, where they overlap, i.e., for values of *R *between 0.05 and 0.06 *μ*m.

Assuming the well established value of bending modulus *k *= 10^-12 ^erg [16], the recalculated coefficients of the surface tension are found in the interval of *σ *~ 0.01-0.02 dyn/cm. These tension values are much smaller comparing to the lipid molecular compressibility (100 dyn/cm) [18] but much larger than the critical surface tension for the instability of the membrane cylinder and "pearling" (10*^-^*^5 ^dyn/cm for (DGDG/DMPC membrane) LNTs of radius *R *~ 0.3 - 5 *μ*m found in [19] work) while comparable with the magnitude of the lateral tension (higher limit) for mutual adhesion of lecithine membranes ~10*^-^*^4 ^erg/cm^2 ^[20].

The small value of the junction radius corresponds to the strongly deformed state of the membrane. These small values should be considered as order estimates, since they are attributes of the assumed toroidal geometry of junctions. The real shape of these junctions is probably more complex and, thus, cannot be described by just two scalar valued parameters. Although freeze-fracture electron microscopy does not reveal bilayers with curvature less than 20 nm, the value *r *~ 1.5 nm which is found from the model is similar to the radius curvature of small inverted pores (for example, it is known that phospholipids spontaneously form inverted membrane structures with the radius varying between 0.5 and 5 nm, and smallest fusion pores have a calculated diameter less than 2.5 nm) [21,22].

## Conclusions

We propose a simple geometrical model for the quantitative explanation of the experimental results on equilibrium geometrical shape and LNTs parameters, *R*(*L*), in the different configurations. The experimental observations show that the nanotube diameter is reduced at shorter lengths and also that the diameter is consistently smaller for the TVC as compared to the TOC for a given length. The observed effect is ascribed to originate from the elastic junctions, since the phenomenon is accentuated in a system containing two necks connected to a vesicle membrane. We approximate the shape of these junctions by simple geometrical shapes and express the free elastic energy of the membrane in terms of the length of the LNT, its radius, the radius of the junction and the tension of the membrane. Variation of the energy with respect to the nanotube radius yields an explicit relation between the radius and the length. The relation is in agreement with observed values. The model enables estimations of the current surface tension coefficient and the curvature at junction regions. The estimated values of the surface tension are of order 10*^-^*^2 ^dyn/cm and the curvature value at junctions are comparable to ones at fusion pores. Furthermore, the proposed model offers a clear explanation of the difference in measurements for TVC and TOC: in contrast to TOC, the TVC features two junction regions, thus, the length of the LNT in this configuration must be twice as long to achieve the same value of the radius.

## Abbreviations

DGDG: digalactosyldiacylglycerol; DMPC: dimyristoylphosphatidylcholine; LNT: lipid nanotube; SBL: soybean liposomes; TOC: tube-only configuration; TVC: two-vesicle configuration.

## Competing interests

The authors declare that they have no competing interests.

## Authors' contributions

RG contributed in development of the geometrical model, analysis of experimental data and participated in writing of the manuscript. MVV participated in the model development and analysis of experimental data, physical interpretation of results and writing the manuscript. KLA and MK have contributed to the experimental part of the study. RK, MK, AGE, and ASC have equally participated in writing of Sections 'Background', 'Experimental', and 'Results and discussion.' RK and MVV provided the idea for the theoretical work. All authors read and approved the final manuscript.
